# Efficacy of low-intensity psychological intervention applied by ICTs for the treatment of depression in primary care: a controlled trial

**DOI:** 10.1186/s12888-015-0475-0

**Published:** 2015-05-07

**Authors:** Adoración Castro, Azucena García-Palacios, Javier García-Campayo, Fermín Mayoral, Cristina Botella, José María García-Herrera, Mari-Cruz Pérez-Yus, Margalida Vives, Rosa M Baños, Miquel Roca, Margalida Gili

**Affiliations:** Institut Universitari d′Investigació en Ciències de la Salut (IUNICS), University of Balearic Islands, Palma de Mallorca, Spain; Red de Investigación en Actividades Preventivas y Promoción de la Salud, Instituto de Salud Carlos III, Madrid, Spain; Department of Clinical and Basic Psychology and Biopsychology, Faculty of Health Sciences, University Jaume I, Castellon, Spain; Department of Psychiatry. Hospital Miguel Servet, University of Zaragoza, Zaragoza, Spain; Mental Health Department, University Regional Hospital of Málaga, Institute of Biomedicine of Málaga, Málaga, Spain; Department of Psychological, Personality, Evaluation and Treatment, University of Valencia, Valencia, Spain; CIBER Fisiopatología Obesidad y Nutrición (CIBERobn), Instituto Salud Carlos III, Madrid, Spain

**Keywords:** Depression, Primary health care, Computer-delivered psychotherapy, Randomized controlled trial

## Abstract

**Background:**

Depression is one of the most common disorders in Psychiatric and Primary Care settings and is associated with significant disability and economic costs. Low-intensity psychological interventions applied by Information and Communication Technologies (ICTs) could be an efficacious and cost-effective therapeutic option for the treatment of depression. The aim of this study is to assess 3 low-intensity psychological interventions applied by ICTs (healthy lifestyle, positive affect and mindfulness) in Primary Care; significant efficacy for depression treatment has previously showed in specialized clinical settings by those interventions, but ICTs were not used.

**Method:**

Multicenter controlled randomized clinical trial in 4 parallel groups. Interventions have been designed and on-line device adaptation has been carried out. Subsequently, the randomized controlled clinical trial will be conducted. A sample of N = 240 mild and moderate depressed patients will be recruited and assessed in Primary Care settings. Patients will be randomly assigned to a) healthy lifestyle psychoeducational program + improved primary care usual treatment (ITAU), b) focused program on positive affect promotion + ITAU c) mindfulness + ITAU or d) ITAU. The intervention format will be one face to face session and four ICTs on-line modules. Patients will be diagnosed with MINI psychiatric interview. Main outcome will be PHQ-9 score. They will be also assessed by SF-12 Health Survey, Client Service Receipt Inventory, EuroQoL-5D questionnaire, Positive and Negative Affect Scale, Five Facet Mindfulness Questionnaire and the Pemberton Happiness Index. Patients will be assessed at baseline, post, 6 and 12 post-treatment months. An intention to treat and per protocol analysis will be performed.

**Discussion:**

Low-intensity psychological interventions applied by Information and Communication Technologies have been not used before in Spain and could be an efficacious and cost-effective therapeutic option for depression treatment. The strength of the study is that it is the first multicenter controlled randomized clinical trial of three low intensity and self-guided interventions applied by ICTs (healthy lifestyle psychoeducational program; focused program on positive affect promotion and brief intervention based on mindfulness) in Primary Care settings.

**Trial registration:**

Current Controlled Trials ISRCTN82388279. Registered 16 April 2014.

## Background

Depression is one of the most common disorders in Psychiatric and Primary Care settings [1] and is associated with significant physical comorbidity [2,3] disability and economic costs [4]. According to the World Health Organization, this disorder will become the second most important cause of disability in 2020 [5]. Previous studies reported that in Spanish Primary Care, depression prevalence is between 13.9 and 29% [1,6] and 60% of patients with depression are attended in a Primary Care Center [7]. Although general practitioners refer only 5-10% of the patients with psychiatric disorders to specialized services [8], mental health services are collapsed all over occidental countries.

There is strong evidence that pharmacotherapy and psychotherapy could be the first-line treatment for depression [9,10]. Although a combination of both have been shown the best option [11], pharmacotherapy is the most common intervention in primary care settings [12], even though relapse is high following cessation, and many patients prefer psychological therapies [13]. In the last decades, there has been a growing interest and commitment to integrate psychotherapy and other mental health services in Primary Care [14]. However, to date this type of treatment is not being used proportionate in Primary Care. Different factors as professional’s training, time needed, costs or work overload, professional’s attitudes and organization, geographical and logistic difficulties may contribute to it. An important goal in Primary Care is that the patient could choose psychological treatment for different reasons: There are patients that would prefer psychotherapy rather than pharmacologic treatment [15,16], and there is a need to offer alternative treatments to the patients that do not respond or respond partially to antidepressants and can benefit from the psychotherapy in terms of costs and relapse prevention [17].

One of the most important difficulties to integrate psychotherapy in Primary Care is lack of time. Many treatment protocols, with empirical support, last one hour weekly during 12-16 sessions. Lack of time and resources suppose important difficulties to the application of this type of therapy. For these reasons, brief psychotherapy for depression is an efficacious alternative [18]. Because of the efficacy of these interventions [10,19,20] and the challenge that depression suppose to the health system, nationals and internationals clinical guides [9,10,21] have proposed a “stepped care” treatment model in Primary Care by which large proportion of patients is treated with significant clinical benefits. This therapy is easier and simpler than formal psychotherapy and it can be used with nontraditional methods such as internet or mobile phones. In depressive patients, low-intensity interventions are recommended to mild and moderate depressive patients. The main interventions are: Cognitive behavior therapy (CBT), Computerized Cognitive Behavior Therapy (CCBT), Self-help programs, Psychoeducational Interventions and mindfulness-based cognitive therapy (MBCT).

Previous research suggests that not all depressed primary care patients are receiving adequate treatment for depression [22] therefore, there is an urgent need to change the way to deliver psychological interventions. There are recommendations regarding going beyond face-to-face psychotherapy in order to improve the accessibility to psychological interventions [23]. In this sense, in the last years, low-intensity psychological interventions applied by Information and Communication Technologies (ICTs) have been shown as an efficacious therapeutic option for the treatment of many mental health problems, including depression. In fact, different meta-analysis have demonstrated that Internet-delivered psychotherapy (iPT) is effective in the treatment of depression [20,24].

Due to the interest about on-line psychotherapy and the need to cover therapeutic management of depressive patients, different European research groups have designed computerized programs for depression prevention and treatment.

Proudfoot and colleagues [25] developed a computerized cognitive behavioral therapy to mild and moderate depression named “Beating the Blues”. It is the most used program and the only computerized treatment program recommended by NICE for the treatment of people with this type of disorder at the moment. Another interactive program based on CCBT is the MoodGym program [26] from the Australian National University, developed to depression prevention [27]. In Spain, there is only one computerized program based on CCBT to depression management in primary care setting [28]. At the present, the study is being conducted by our research group and results will be presented soon.

Considering that low-intensity psychological interventions applied by Information and Communication Technologies (ICTs) could be an efficacious and cost-effective therapeutic option for the treatment of depression, the aim of the present study will be to assess three low-intensity psychological interventions (healthy lifestyle psychoeducational program, focused program on positive affect promotion and brief intervention based on mindfulness) applied by ICTs in Primary Care.

## Methods

### Study design

This is a multicenter controlled randomized clinical trial in four parallel groups: a) healthy lifestyle psychoeducational program, b) focused program on positive affect promotion, c) brief intervention based on mindfulness and d) improved treatment as usual (ITAU) group in primary care.

### Setting and study sample

The recruitment strategy will be performed by General Practitioners (GPs) from primary health centers of the four Spanish regions involved in the study (Andalucía, Aragon, Islas Baleares and Comunidad Valenciana). Inclusion criteria are: older than 18 years, DSM-5 diagnose of Major Depression or Dysthymia, mild or moderate depression expressed as score lower than 14 in the Patient Health Questionnaire (PHQ) [29], depressive symptoms presented for at least two months, be able to read and understand Spanish language and enough capacity for understanding and signing the written informed consent form. Exclusion criteria includes any diagnose of disease that may affect central nervous system (brain pathology, traumatic brain injury, dementia, etc.), any psychiatric disorder other than Major Depression, Dysthymia, anxiety disorders or personality disorders, any medical, infectious or degenerative disease that may affect mood, presence of delusional ideas or hallucinations consistent or not with mood, and suicide risk.

### Sample size

According to the literature, with a standard deviation (SD) of 9.2 and a mean of 16.2 in the control group (ITAU) [30], 14.59 in the positive affect group [31], 16.12 in the healthy lifestyle group [32] and 10.3 in the mindfulness group [30], accepting an alpha of 0.05 and a beta risk < 0.2 in a bilateral contrast, and assuming a 25% patient loss to follow-up, we need 60 subjects in each condition. Therefore, the total sample size was determined at 240. This sample size also allows calculating clinically significant difference in the main outcome variable, PHQ-9 [29], and this difference has been placed at 5 points.

### Recruitment

The recruitment strategy will be performed in primary care settings by participating GPs among patients fulfilling study criteria. When the GP identifies a potential participant, he/she will explain the patient the characteristics of the study. Whether the patient is interested in participating he/she will sign an informed consent and the GP will fill a referral form describing the sociodemographic characteristics of the patient and a checklist for inclusion/exclusion criteria, and will give him/her the patient’s information sheet and a handout describing the study. If the patient does not want to participate or does not meet inclusion criteria, the GP will fill the referral form describing the reason for not including him/her. The GP will send these documents by fax to the local researcher. Subjects will be interviewed in the next 3 days by the researcher. After confirming that they have signed the informed consent and have understood the study and the treatment options, the researcher will administer the psychological assessment instruments related with inclusion criteria: MINI International Neuropsychiatric Interview [33], PHQ-9 [29] and sociodemographic variables. The researcher will collect baseline data and will contact an independent researcher to implement the randomization.

### Randomization, allocation and masking of study groups

Each patient will be allocated to either one of the four groups using a computer-generated random number sequence. We will use block allocation, with 4 blocks (one for each participating region) including 60 patients per block, with about 60 patients per arm. The allocation will be carried out by an independent researcher, belonging to REDIAPP (Research Network on Preventative Activities and Health Promotion), who is not involved in the study. The method used to implement the random allocation sequence will be a central telephone. The sequence will be concealed until interventions are assigned. Patients will agree to participate before the random allocation and without knowing which treatment they will be allocated to. Study personnel conducting psychological assessments will be masked to participants’ treatment conditions.

**Figure 1:**
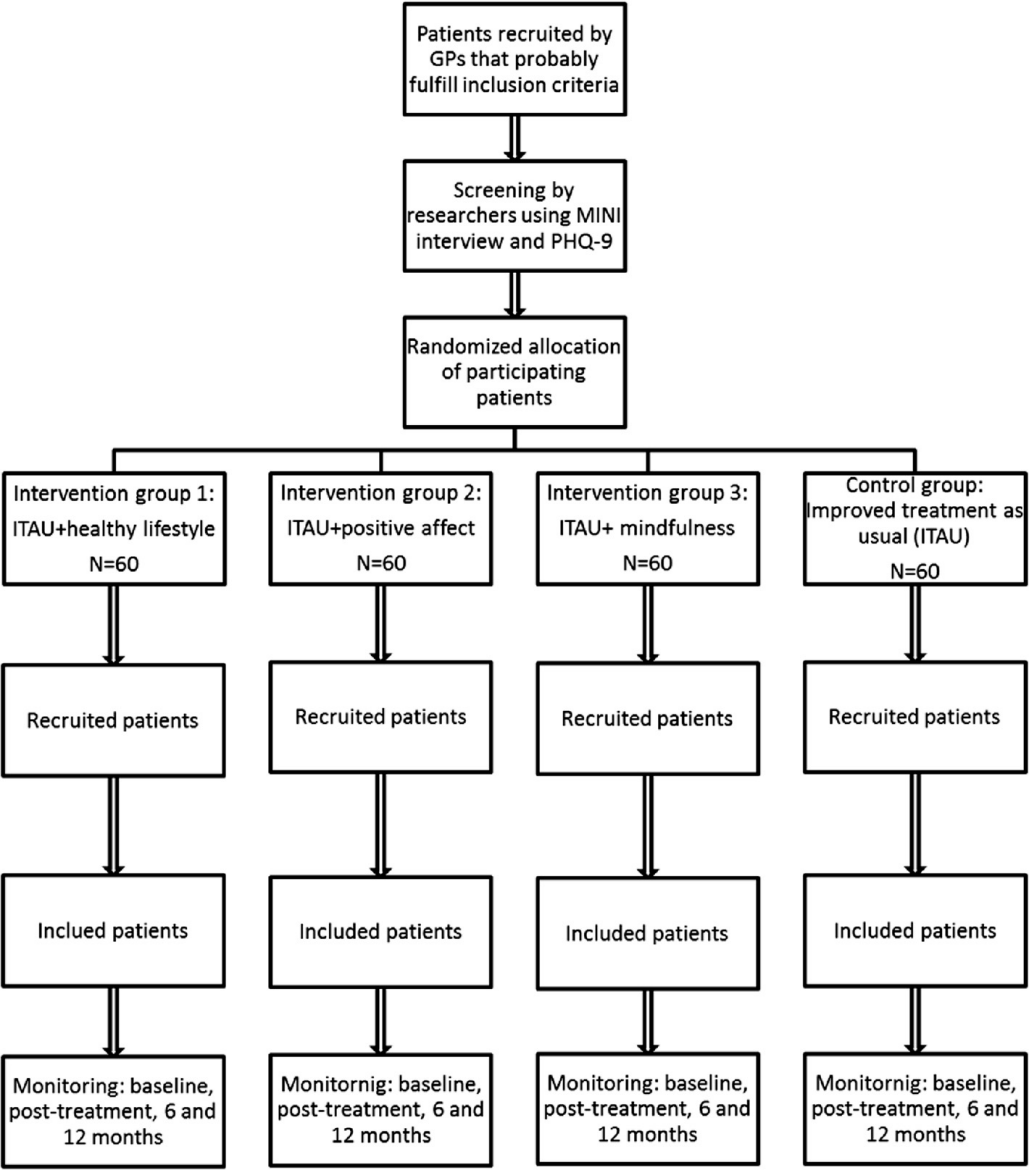
Flowchart of the study: Randomization, sampling and monitoring of patients.

The researcher that administers baseline assessments will be unaware of the treatment group to which the patient belongs to. This researcher will be different from the one that administer the questionnaires over the study. GPs will be also unaware, as long as possible, of the arm to which the patient has been randomized, since their treatment should be exclusively based on the recommendations of the guides for the treatment of depression. The flowchart of the study is summarized in Figure 1.

### Interventions

All interventions (except ITAU) are composed of one face-to-face group session and 4 online, individual and interactive therapeutic modules.

Face-to-face session will involve 3-5 patients and will be 90 minutes in length. The aim of this session will be to explain the program structure and main components of treatment, and to motivate participants for change.

The online therapeutic modules are oriented to work on different psychological techniques and the duration of each module will be approximately 60 minutes. All modules include an explanation of the module contents, check questions to test if they understand the contents and exercises to practice such techniques. These modules are sequential, in order to move step by step, all along the program. However, user can review the module contents once they are finished. Duration of the program can vary among users, it is estimated the duration for most people will be 4-8 weeks. The participants will receive two weekly-automated mobile phone messages, encouraging them to proceed with the program and reminding them of the importance of doing the tasks in each module. Furthermore, the participants will receive an automated e-mail encouraging them to continue with the modules if they have not accessed the program for a week. The program also offers continued feedback to users through the assessment tools showing them their progress throughout the entire treatment process.

All groups of patients will receive treatment as usual improved by their GPs, as described below in this section. All groups of patients will also receive a participant manual with information about technical aspects of the online program.

#### Psychoeducational program for the promotion of a healthy lifestyle

This program provides information to understand the relationship between physical and mental health and physical activity, diet and sleep control.

The content of the program is the following:

- ***M1****.****Beginning of a lifestyle change***: This module aims to teach the importance of healthy lifestyle to improve emotional health and general well-being and to give structured hygienic-dietary recommendations. The patient will learn to identify both healthy and risk behaviors, and to recognize the most common obstacles that prevent the depressed patients from adopting a healthy lifestyle.
- ***M2****.****Physical activity****.****Learning to move on****:* This module focuses on behavior activation by teaching the importance of “moving on” and taking regular exercise to improve mood. In this module the main aim is to give information about the most recommended exercises to improve mood, and to train the patient in learning procedures to increase the motivation, to start being more active, and to maintain this physical regularly.
- ***M3****.****Diet****.****Learning to eat****:* This module is devoted to teach the importance of diet to achieve a good physical and mental health, and the role of the Mediterranean diet in the prevention and treatment of depression. For this purpose the obstacles to maintain a healthy diet will be analyzed.
- ***M4****.****Sleep****.****The importance of good sleep****:* This module provides information to understand the relationship between sleep and general health. The patient will be trained to develop strategies to improve sleep.

#### Psychological intervention for the promotion of positive affect

The purpose of this program is to decrease depressive symptomatology and to prevent relapses using the promotion of wellbeing and positive affect.

The content of the program is the following:

- ***M1****.****Learning to live****:* The first module is dedicated to the importance of establishing and maintaining an adequate activity level and the relevance of choosing activities which are significant, with a personal meaning for the individual. The content will cover also the importance of social support in order to make easier the task of establishing meaningful activities. The patient will be trained to carry out and monitor meaningful activities using a therapeutic tool, the activity diary.
- ***M2****.****Learning to enjoy****:* In this module the main aim is to give education about the effect of positive emotions and to train the patient in learning procedures to increase the likelihood of experiencing positive emotions, promoting the occurrence of pleasant activities in order to learn to enjoy the present moment.
- ***M3****.****Accepting to live****:* In the previous module the focus was in positive emotions related with the present time. In this module the patient will be trained in focusing in positive emotions related with the past (like gratitude) or the future (like optimism). This module also deals with the identification and management of beliefs and behaviors that disturb the good moments.
- ***M4****.****Living and learning****:* The aim of this module is to train the patient in understanding life as a continuous process of learning and personal growth, emphasizing the training in strategies to promote psychological strengths, resilience and meaningful goals linked to important values. The content of the module will also include understanding the effort needed in order to achieve a meaningful life, helping the patient to make a plan for the future to achieve his/her life goals.

#### Brief intervention based on mindfulness

This program, which aim is to decrease depressive symptomatology using mindfulness, emphasizing the benefits of this therapy.

The content of the program is the following:

- ***M1****.****Getting to know mindfulness***: This module aims to show what mindfulness is, prejudices about it, the inattention problem and some of its main benefits and recommendations to practice it. Finally, a mindful practice (the raisin exercise) will be shown.
- ***M2****.****Establishing formal and informal practices****:* In this module it will be raised the importance of the establishment not only of formal but also of informal practice. For this purpose different techniques such as the three minutes practice will be shown.
- ***M3****.****Thought management****,****body scan practice and values****:* This module aims to help people to see the importance of values to keep a regular mindfulness practice. It also will include the body scan practice.
- ***M4****.****Self****-****compassion****.****Integrating mindfulness in everyday life****:* This module will help people to establish a regular practice of mindfulness to be indefinitely kept. It also will include a very powerful practice, self-compassion, which has shown efficacy by itself [27].

#### Improved treatment as usual (ITAU)

All the patients included in the study (whatever the treatment group randomly assigned) will be also treated by their GPs. The Treatment as Usual in primary care will be improved because the participating GP will receive a training program on a widely used Spanish Guide for the Treatment of Depression in Primary Care [7]. In case of suicide risk, severe social dysfunction or worsening of symptoms, it is recommended to refer the patient to mental health facilities. In practice, ITAU in primary care is any kind of treatment administered by the GP to the patient with depression.

### Instruments

Patients will be assessed at baseline, post-treatment, and 6 and 12 post-treatment months. The study variables assessed are summarized in Table 1.

**Table 1:** **Study variables**

**Instrument**	**Assessment area**	**Time of assessment**	**Applied by**
MINI Neuropsychiatric interview	Psychiatric diagnosis	Baseline	Researcher A
PHQ-9	Severity of depression	Baseline and follow-up sessions*	Researcher A (baseline) Online (follow-up sessions)
Sociodemographic data	Gender, age, marital status, education, occupation, economical level	Baseline	Researcher A
SF-12 Health Survey	Health-related quality of life	Baseline and follow-up sessions*	Online
CRSI	Health and social services use	Baseline and 6 and 12 post-treatment months	Researcher A (baseline) Researcher B (6 and 12 post-treatment months)
EQ-5D	Health related quality of life	Baseline and 6 and 12 post-treatment months	Online
PANAS	Positive and negative affect	Baseline and 6 and 12 post-treatment months	Online
FFMQ	Facets and factors of mindfulness	Baseline and 6 and 12 post-treatment months	Online
PHI	Remembered and experienced well-being	Baseline and 6 and 12 post-treatment months	Online

*Follow-up sessions: Post-treatment, 6 and 12 post-treatment months.

#### Main outcome

The primary outcome will be depressive symptom severity assessed via PHQ-9 [34] using the Spanish validated version [29]. This is an instrument for diagnosis and measuring the severity of depression. PHQ-9 is brief and it is completed by the patient. It will be registered at four times: baseline, post-treatment, 6 month follow-up, and 12 month follow-up.

#### Secondary outcomes

Sociodemographic variables. Relevant sociodemographic information regarding gender, age, marital status (single, married/relationship, separated/divorced, and widowed), education (years of education), occupation, economical level will be collected.

Mini-International Neuropsychiatric Interview (MINI). This is a short structured diagnostic psychiatric interview that yields key DSM-IV and ICD-10 diagnoses [35]. MINI can be administered in a short period of time and clinical interviewers need only a brief training. It will be registered at baseline using its Spanish validated version [33].

SF-12 Health Survey [36]. This instrument consists of 12 items that measure the health-related quality of life. A psychometric analysis suggested that it has adequate psychometric properties and a validated Spanish version [37].

Client Service Receipt Inventory [38] (CSRI-Spanish version) [39]. Questionnaire for collecting information about use of healthcare and social care services and other economic impacts (such as time off work due to illness). The variant used in this study was designed to collect retrospective data on service utilization during the previous months after the last assessment. Data on baseline assess the previous six months before inclusion.

EuroQoL-5D questionnaire (EQ-5D – Spanish version) [40]. Generic instrument of health related quality of life. It has two parts: Part 1 records self-reported problems in each of five domains: mobility, self-care, usual activities, pain/discomfort and anxiety/depression. Each domain is divided into three levels of severity corresponding to no problems, some problems and extreme problems, which allows obtaining a population-based preference score or societal index (SI). A total of 243 theoretically possible health states can be obtained and the SI is calculated on the basis of these health states. Values range from 1 (best health state) to 0 (death). However, this index may also provide negative values that correspond to health states perceived as worse than death-. Utility scores for these health states were assigned using the readily available Spanish population tariffs [41]. Part 2 records the subject’s self-assessed health on a VAS, a 10 cm vertical line on which the best and worst imaginable health states score 100 and 0 respectively.

Positive and Negative Affect Scale (PANAS) [42] PANAS consists of 20 items that evaluate two independent dimensions: positive affect (PA) and negative effect (NA). The range for each scale [10 items on each] is 10 to 50. The instrument’s psychometric properties are quite satisfactory. It has a validated Spanish version [43].

Five Facet Mindfulness Questionnaire (FFMQ) [44]: This questionnaire comprises 39 items that assess five facets or factors of mindfulness: observing (8 items), describing (8 items), and acting with awareness (8 items), not judging inner experience (8 items) and not reacting to inner experience (7 items). Items are rated using a Likert scale that ranges from 1 (“never or very rarely true”) to 5 (“very often or always true”). The Spanish version of this scale has been validated [45] and has been shown to have good internal consistency and reliability.

The Pemberton Happiness Index (PHI) [46]. This instrument consists of eleven items related to different domains of remembered well-being (general, hedonic, eudaimonic and social well-being) and ten items related to experienced well-being (positive and negative emotional events that possibly happened the day before). A psychometric analysis suggested that it has adequate psychometric properties and a validated Spanish version [46].

### Ethical aspects

A general overview of the aims and characteristics of the study and the interventions will be provided to the patients. They will also be informed that they will be participating voluntarily, and that they can choose to withdraw at any time with the guarantee that they will continue to receive the treatment considered most appropriate by their GP. Informed consent will be obtained from the participants before they are aware of which group they are to be included in. For ethical reasons, patients allocated to Treatment as Usual will be offered the possibility to complete the psychotherapy program. The study follows national and international norms (Helsinki Convention and the Declaration of Madrid of the World Psychiatric Association). The Study Protocol was approved by the Comité d’Ètica de la Investigació de les Illes Balears (CEI-IB) (ref: IB 2144/13 PI).

An important issue in a study using ICTs is participants’ data protection. In order to protect personal information, AES (*Advanced Encryption Standard*) strategies regarding the use of personal passwords and data encryption will be followed.

### Analysis strategy

#### Analysis of clinical efficacy

Intention-to-treat and per-protocol analysis will be used. Analysis will include the description and “head-to head” comparison between the four groups. Descriptive statistics of the included variables (mean and 95 per cent confidence interval for normally distributed quantitative variables; and median and interquartile range for abnormally distributed quantitative variables) will be performed. To confirm main hypothesis, all variables will be compared (t0-tk) using an analysis of variance (ANOVA) and post-hoc tests or Kruskal-Wallis non-parametric tests. Finally, more sophisticated multivariate analysis, including multilevel regression, will be used. The effect size of improvement and intention-to-treat (ITT) will be estimated.

#### Descriptions of costing procedure

Data collection on the use and utilization of health and social services will be collected through the questionnaire Client Service Receipt Inventory (CSRI) Spanish version [40]. The variant to be used in this study was designed to retrospectively collect data on service use during the previous 6 months.

Costs were estimated from the healthcare and societal perspectives during the six months of follow-up. Direct health care costs were calculated by adding the costs derived from medication consumption, medical tests, use of health-related services, and cost of the staff running the CBT intervention.

The cost of medication will be calculated by determining the price per milligram according to the Databases of the drug from the Ministry of Health and Consumer and included the value-added tax. The total costs of medications were calculated by multiplying the price per milligram by the daily dosage used (in milligrams) and the number of days that the treatment was received. The main source of the unit cost data for medical tests and health services will be use the OBLIKUE database of health care costs. The OBLIKUE database contains information about Spanish healthcare service costs and was derived by systematic reviews of the literature; it consists of approximately 18,000 entries.

Indirect costs (lost productivity): Lost productivity will be calculated using the human capital approach, which involves multiplying the minimum daily wage in Spain for 2014 by the number of days of sick leave, as reported by each patient. Finally, total costs were calculated by adding the direct and indirect costs. Unit costs are expressed in Euros (€) based on 2014 prices.

In our study the general guidelines for conducting pharmacoeconomic analyzes in Spain and the International Society for Pharmacoeconomics and Outcomes Research (ISPOR) [47,48] will be followed.

#### Utility scores

Utility scores will be obtained from the questionnaires quality of life related to health EuroQol 5D and SF-12. The EQ-5D is used to rate patients’ quality of life on a scale from 0 (as bad as death) to 1 (perfect health). Negative values are possible and indicate a health state that is “worse than death.” QALYs were calculated on the basis of these scores using the Spanish tariffs of EQ-5D [49]. Along with EQ-5D utility scores, scores recorded on the EQ VAS were also used as an outcome for the analysis.

##### Analysis perspective and time horizon

The perspective used in our analysis will be twofold: on one hand the social perspective, to include all costs and quality of life and moreover the perspective of the national health system.

The time horizon of the study is 12 months, so there will have to use any kind of discount or costs or profits.

#### Cost-utility analysis

Cost-utility will be explored through the calculation of incremental cost-effectiveness ratios (ICER), defined as the ratio between incremental costs and incremental effects measured on QALYs or EQ VAS [50]. QALYs were approximated by using the area under-the-curve technique.

To estimate the incremental cost effectiveness ratio - ICER (marginal incremental cost and incremental effects) the following equation is used:

$$ ICER=\frac{Cost\  strategy\ 1- Cost\  strategy\ 2}{effectiveness\  strategy\ 1- effectiveness\  strategy\ 2} $$

Cost-effectiveness acceptability curves (CEACs) will also be plotted when necessary [50]. CEACs display the probability that the intervention is cost-effective, given a varying threshold for the willingness to pay for each QALY gained. The curves obtained incorporate the uncertainty that exists around the estimates of incremental costs and incremental effects associated with the intervention [51].

Data collection will be performed using EXCEL software and then for statistical analysis we used the SPSS (SPSS Inc., Chicago, IL, USA) software version 20, licensed for University of Malaga.

Frequency and proportions used for descriptive statistics of categorical or qualitative variables. For quantitative variables the mean and standard deviation were obtained.

For inferential statistical test Chi-square test for qualitative variables for qualitative and quantitative variables was used test one-way ANOVA, and for the test of the T-Student quantitative variables. In all cases statistical significance corresponded to a value of p <0.05.

### Forecast execution dates

Initial recruitment of patients: February 2015

Finalization of patient recruitment: July 2015

Finalization of patient monitoring period: July 2016

Publication of results: December 2016

## Discussion

Brief psychotherapy is an effective alternative for depression treatment to offer in primary care [18]. Moreover, the effectiveness of Internet-delivered psychotherapy for this disorder has also been demonstrated [24]. Low-intensity psychological interventions applied by Information and Communication Technologies (ICTs) are not frequently used in Spain and could be an efficacious and cost-effective therapeutic option for the treatment of depression. The strength of the study is that, to our knowledge, this is the first multicenter controlled randomized clinical trial of four low intensity and self-guided interventions applied by ICTs (healthy lifestyle psychoeducational program; focused program on positive affect promotion; brief intervention based on mindfulness; and improved treatment as usual group) in Primary Care settings.

Two important limitations of the study will be the negative attitudes from GP to recommend this treatment and dropout rates in treatment groups (significantly greater than in the control group according to previous studies). Efforts to maintain dropout rates in the range of 30% and training sessions for participating GPs will be carried out to overcome these limitations.

### Clinical implications

This is probably the first study in Spain aiming to improve symptomatology and quality of life of depressed patients using low intensity interventions applied by ICTs. The treatment programs used in this study includes therapeutic strategies (mindfulness, healthy lifestyle and positive affect) that have proven their efficacy for depression treatment, nerveless it is the first study that adapts these interventions to ICTs.

Efficiency and efficacy of these interventions could be suitable for implementation from an economic point of view. Moreover, they could be used in problems were depression or adjustment disorders are comorbid or other emotional disorders are presented. In conclusion, positive results of this study could have a significant impact on the society.

## Abbreviations

ICTs: Information and communication technologies
ITAU: Improved primary care usual treatment
CBT: Cognitive behavior therapy
CCBT: Computerized cognitive behavior therapy
MBCT: Mindfulness-based cognitive therapy
iPT: Internet-delivered psychotherapy
GP: General practitioners
PHQ: Patient health questionnaire
REDIAPP: Research Network on Preventative Activities and Health Promotion
MINI: Mini-International Neuropsychiatric Interview
CSRI: Client service receipt inventory
PANAS: Positive and negative affect scale
FFMQ: Five facet mindfulness questionnaire
PHI: The Pemberton happiness index
ISPOR: International Society for Pharmacoeconomics and Outcomes Research
ICER: Incremental cost-effectiveness ratios
